# Cognitive Bias in Ambiguity Judgements: Using Computational Models to Dissect the Effects of Mild Mood Manipulation in Humans

**DOI:** 10.1371/journal.pone.0165840

**Published:** 2016-11-09

**Authors:** Kiyohito Iigaya, Aurelie Jolivald, Wittawat Jitkrittum, Iain D. Gilchrist, Peter Dayan, Elizabeth Paul, Michael Mendl

**Affiliations:** 1 Gatsby Computational Neuroscience Unit, UCL, London W1T 4JG, United Kingdom; 2 Centre for Behavioural Biology, School of Veterinary Science, University of Bristol, Langford, BS40 5DU, United Kingdom; 3 School of Experimental Psychology, University of Bristol, Bristol, BS8 1TU, United Kingdom; Universita degli Studi di Bologna, ITALY

## Abstract

Positive and negative moods can be treated as prior expectations over future delivery of rewards and punishments. This provides an inferential foundation for the cognitive (judgement) bias task, now widely-used for assessing affective states in non-human animals. In the task, information about affect is extracted from the optimistic or pessimistic manner in which participants resolve ambiguities in sensory input. Here, we report a novel variant of the task aimed at dissecting the effects of affect manipulations on perceptual and value computations for decision-making under ambiguity in humans. Participants were instructed to judge which way a Gabor patch (250ms presentation) was leaning. If the stimulus leant one way (e.g. left), pressing the REWard key yielded a monetary WIN whilst pressing the SAFE key failed to acquire the WIN. If it leant the other way (e.g. right), pressing the SAFE key avoided a LOSS whilst pressing the REWard key incurred the LOSS. The size (0–100 UK pence) of the offered WIN and threatened LOSS, and the ambiguity of the stimulus (vertical being completely ambiguous) were varied on a trial-by-trial basis, allowing us to investigate how decisions were affected by differing combinations of these factors. Half the subjects performed the task in a ‘Pleasantly’ decorated room and were given a gift (bag of sweets) prior to starting, whilst the other half were in a bare ‘Unpleasant’ room and were not given anything. Although these treatments had little effect on self-reported mood, they did lead to differences in decision-making. All subjects were risk averse under ambiguity, consistent with the notion of loss aversion. Analysis using a Bayesian decision model indicated that Unpleasant Room subjects were (‘pessimistically’) biased towards choosing the SAFE key under ambiguity, but also weighed WINS more heavily than LOSSes compared to Pleasant Room subjects. These apparently contradictory findings may be explained by the influence of affect on different processes underlying decision-making, and the task presented here offers opportunities for further dissecting such processes.

## Introduction

It is well established that emotional states influence the way that we process sensory stimuli, and thereby the decisions we make based on those stimuli. For example, people experiencing negative affective states, especially anxiety, are more likely than happier people to attend to threatening stimuli and make negative interpretations of ambiguous stimuli and situations [1–3]. Depressed people also have difficulty disengaging attention from threatening stimuli [4]; and both anxious and depressed people tend to recall negative memories and make more pessimistic predictions about the future [5]. There are suggestions that positive affective states have the reverse effects, although there is a dearth of data in this area. Overall, although there are exceptions and specificities [5], negatively and positively valenced affective states seem to be associated respectively with negatively and positively biased information processing.

These links between affect and decision-making inspired us to develop a non-linguistic task that used cognition and choice as an indicator of affective state in humans and other animals [6]. For instance, in the original variant of this task, rats were trained to make a ‘positive’ (P) response (lever press) when one tone was sounded in order to acquire a food reward, and a ‘negative’ (N) response (no lever press) when a different tone was sounded in order to avoid a burst of white noise [7]. Once trained on this conditional discrimination task, ‘ambiguous’ tones of intermediate frequency were occasionally presented during training sessions to investigate whether the animals made response P, indicating anticipation of food (operationally defined as an ‘optimistic’ decision (see [8]), or response N, indicating anticipation of the possibility of noise (a ‘pessimistic’ decision). Negative mood would favour cautious or pessimistic decisions under ambiguity [9], and so could be determined from a bias towards N. This could be validated, for instance, by examining the consequence in the task of the regular experience of negative events. For example, in Harding et al.’s study [7], repeated experience of mildly aversive unpredictable events resulted in a pessimistic bias as predicted.

The task, often referred to as a ‘judgement bias task’, has now been used in over 75 published studies on a variety of species [10–14]. There is substantial evidence in concordance with our interpretation, suggesting that the task is a promising measure of animal affective state. It also works well in humans, thereby tying studies which employ linguistic methods including questionnaires and homophone and ambiguous sentence interpretation, to tasks that can be applied to non-linguistic subjects. For instance, several studies have used the task in people to reveal ‘pessimistic’ responses to ambiguity in subjects reporting high negative activation and low positive activation PANAS scores [15], high STAI state anxiety and visual analogue scale anxiety ratings [16], and high ‘reflective pondering’ scores [17]. In a related paradigm, patients with panic disorder showed a less steep generalisation curve to cues close to a cue predicting electric shock than did healthy controls [18].

However, along with some null results and opposite findings, various questions remain to be answered, notably exactly how affect influences different components of the process of decision-making. Answering this requires a more comprehensive theoretical treatment. We adopt Bayesian decision theory [19], in which subjects make choices that maximize the utility that can be expected based on conclusions that are drawn about the state of the world by combining prior beliefs about the environment (priors) with current observations (likelihoods). In these terms, there are two not-mutually-exclusive ways that affective states (especially long-term mood states) could guide decisions under ambiguity. One is by influencing the prior probabilities of positive and negative events in the environment. The second is by altering the utility function, i.e., the valuation of rewards and punishments, as in the case of dysphoric anehdonia [20]. Indeed, the interplay between such effects could underlie the null or opposite findings of some animal studies.

Investigating and distinguishing these possibilities is challenging. One approach is to design tasks which provide trial-by-trial variation in a number of features (e.g. degree of cue ambiguity; size of reward and punishment) such that decisions made in each trial can be used to evaluate how interpretation of these features is influenced by the subjects’ affective states. Here, we implement this approach in human subjects. We fit a Bayesian decision-theoretic model of the behaviour to extract parameters characterizing the various components of choice. By exposing subjects to one of two affect manipulation conditions intended to generate either a positive or negative affective state, we investigate whether model parameter values (e.g. bias in decision curves, relative weighting of rewarded vs punished outcomes) differ between the two conditions. In this way, we seek to disentangle aspects of the influence of affect on different components of the processes of decision-making. Throughout, we use the term ‘ambiguity’ to refer to the uncertainty of both the meaning of the presented stimuli and the probability of their outcomes. ‘Risk’ on the other hand is used to refer to the variation in probability of rewarding or punishing outcomes generated by each response; thus ‘risk aversion’ involves selecting the response which has the lowest variation in outcome probabilities.

## Materials and Methods

### Experiments

#### Participants

Forty-eight participants from the University of Bristol community were included in the study. Their average age was 20.6 years (SD = 5.9) years; 62% were female, 83% were right-handed, and 96% were native English speakers.

#### Ethics

Ethical approval was provided by the University of Bristol Faculty of Medical and Veterinary Sciences Committee for Research Ethics (Approval number: 5581). All participants gave written informed consent to take part in the study, and were debriefed following the experiment. Data were stored anonymously.

#### Task

**Fig 1:**
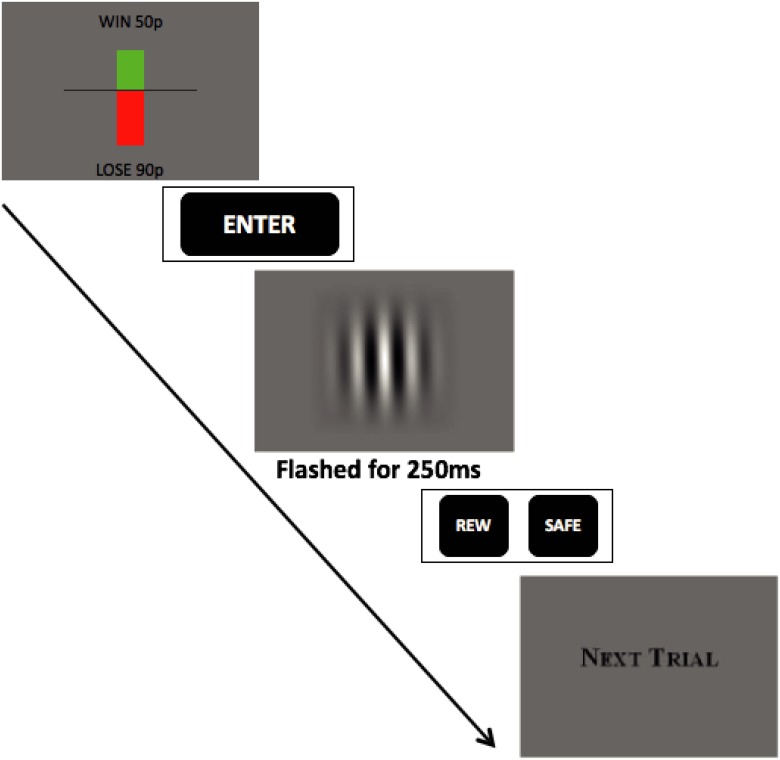
The judgement bias task. Each trial started with presentation of the trial-specific offered win (indicated by the green bar) and threatened loss (red bar). Subjects studied this information and then pressed the ‘Enter’ key. A Gabor patch stimulus was then flashed on the screen for 250ms. The subject had to decide which way the patch was leaning and press either the REW or SAFE key as appropriate. The next trial then followed. See text for details.

The task was presented on an Iiyama vision master pro 454 HM903DT B 19 inch Diamondtron CRT monitor, and written in Matlab^™^ (The Mathworks Inc., Natick, MA) using the PsychToolBox extensions. Participants were seated 57cm from the screen, and their eyes were level with its centre. The start of a trial was indicated by a display of the offered reward and threatened punishment on that trial, denoted by the length of vertical bars emanating from a horizontal line at the centre of the screen. A green bar rose from this line to indicate the size of reward on offer; and a red bar dropped below the line to indicate the size of threatened punishment. Text (‘WIN xx p’ above the green bar; ‘LOSE yy p’ below the red bar) indicated the actual amount of money (0–100 UK pence) that could be won or lost on that trial (Fig 1).

When a participant pressed the ‘Enter’ key (there was no time limit for this) a stimulus (Gabor patch) appeared for 250ms. The participant then had to report, without time limitation, whether the stimulus was tilted towards the side that predicted reward or the side that predicted punishment by pressing one of two keys. For half the participants, a stimulus leaning left predicted a reward (a win of *R*^+^ ∈ [0, 100] UK pence); this was called the WIN side. Conversely, a stimulus leaning right predicted a punishment (a loss of *R*^−^ ∈ [0, 100] UK pence); this was called the LOSS side. The opposite was true for the other participants.

If the stimulus actually leant towards the WIN side, participants needed to press the REW key to obtain the offered reward for that trial. However, if the stimulus leant towards the LOSS side, they needed to press the SAFE key to avoid the threatened punishment and losing the denoted amount of money for that trial. If participants pressed the SAFE key when the stimulus leant towards the WIN side, they failed to win the reward on offer for that trial. And if they pressed the REW key when the stimulus leant towards the LOSS side, they incurred the threatened punishment and lost money. Thus we refer to REW as the *risky* key or choice (as subjects could win or lose) whilst SAFE is the *safe* key or choice (as the worst outcome is 0). Subjects were told that for each trial, based on how sure they were that the image was leaning to one side or the other *and* how much was at stake, their job was to choose either the REW or SAFE key. After choosing a key, the next trial commenced immediately (see Fig 1).

#### Stimuli

The stimuli were Gabor patches that had a contrast close to 100 percent. The standard deviation of the Gaussian envelope was 2 degrees and the grating had a spatial frequency of 0.5 cycles/degree. In practice this meant that the Gabor patch has a visible spatial size of about 12 degrees or 12 cm at this viewing distance. The background of the display was set at the mean of the luminance of the Gabor patch which matched the edges of the Gaussian envelope of the patches. A spirit level was used to ensure that the monitor was exactly horizontal before tests.

In the task, the patches were rotated either to the left or the right, by a maximum of 1.4 degrees from vertical. The orientation of the stimulus was coded using a variable (ambiguity) that varied from -1 (maximum angle to the LOSS side) to 1 (maximum angle to the WIN side). ‘Ambiguous’ stimuli deemed hard to classify were those with orientations ranging from -0.25 to 0.25 (a range of 0.35 degrees around the vertical). This was based on a pilot study which used the same methods to present a different set of subjects with a range of stimuli that they simply had to classify as leaning to the left or right. The proportion of Gabor patches presented for 250ms and tilted at up to 0.35 degrees from the vertical that subjects were able to correctly classify did not differ from chance. However, subjects were able to classify Gabor patches tilted away from the vertical by between 0.35–1.4 degrees better than would be expected by chance.

#### Experimental procedure

**Fig 2:**
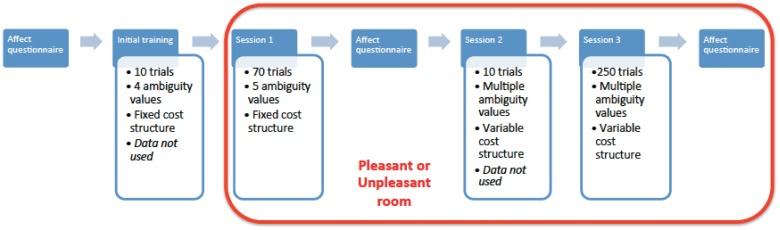
Schematic of the experimental procedure. The order in which affect questionnaires and the different phases of task training and testing were carried out is shown, together with basic information on the trial structure of each session. Phases within the red box took place in either the Pleasant or Unpleasant room. See text for more details.

The schematic of the experimental procedure is shown in Fig 2. At the start of the session, which lasted roughly one hour, subjects were asked to complete two psychometric scales to measure current mood. The Affect Grid [21] was a 9*9 grid on which subjects were asked to mark their mood along two axes (from very deactivated to very activated and from very negative to very positive), and the Positive and Negative Affect Schedule (PANAS [22]) was a list of 20 mood terms that subjects had to rate in term of relevance to their mood at the moment of the test.

Subjects were then given standardised instructions for the decision making-task. During recruitment they had been informed that they would be paid to participate in the study, and they were now offered a baseline monetary compensation of £7, and up to £7 extra based on their performance in the task. To motivate performance across the experiment, subjects were told that they would receive the actual monetary outcome for a randomly chosen 10 trials during test Sessions 1 and 3 (see below) and that this would be added to, or taken away from, their initial monetary compensation of £7. Participants then completed a 10-trial practice session in which the cost structure of the task was fixed, with offered rewards (WIN) and threatened punishments (LOSE) of 50p for all trials. They received immediate feedback on their performance after making each choice. In this session, four values of ambiguity were used: -1, -.25, .25 and 1. The practice session and Session 1 used a fixed-cost structure because pilot work showed that subjects picked up the task better if the different elements (perceptual ambiguity; varying cost structure) were introduced separately. A fixed cost structure is also typical of animal versions of the task making Session 1 more directly comparable to these than the varying cost structure of Session 3 which was designed to tease apart the influence of affect on different aspects of the decision process.

Following the practice session, and to manipulate their emotional state, participants were led up a flight of stairs to one of two rooms: a ‘Pleasant Room’ decorated with daffodils and pictures of flowers (N = 24), and an ‘Unpleasant Room’ left bare (N = 24). Subjects in the Pleasant Room were given a bag of sweets at this stage [23] whilst those in the Unpleasant Room received the sweets during the debrief at the end of the experiment. In order to minimise any explicit awareness of the affect manipulations, subjects were not informed about the presence of the room to which they were not assigned. The general layout of both rooms was similar. We sometimes refer to the participants according to the room to which they were assigned.

Participants then completed the three remaining parts of the decision-making task. Session 1 (70 trials) involved 5 different levels of ambiguity (-1, -.25, 0, .25, 1), with the same cost structure as in the Practice Session (i.e. WIN 50p or LOSE 50p). No feedback was provided. In Session 2 (10 trials), trial-by-trial alterations in the cost structure were introduced (WIN and LOSE were different on each trial) in order to train subjects that WIN and LOSE could vary as well as ambiguity. Feedback was provided after each trial. In Session 3 (250 trials) the combination of ambiguity, WIN and LOSE varied randomly from trial to trial, with 70 percent of stimuli being in the difficult to classify ‘ambiguous’ range (-.25 to .25). All subjects received the same sequence of combinations which were drawn using the rand function in Matlab. Roughly one quarter of trials came from each quadrant of the cost structure distribution (i.e. WIN 0–50p and LOSE 0–50P; WIN 0–50P and LOSE 60–100p; WIN 60–100P and LOSE 0–50p; WIN 60–100p and LOSE 60–100p), and each cost structure combination was presented twice, once with an ambiguity value of *x* and once with a value of *-x*. This ensured that subjects from both the ‘left-lean predicts reward’ and ‘right-lean predicts reward’ groups received the same stimuli. No feedback was provided. Performance in Sessions 1 and 3 were analysed as described below.

During the task, lights were turned off to prevent subjects using vertical cues in the room to solve the task. In addition to answering psychological questionnaires at the start of the experiment, subjects also filled in the Affect Grid and PANAS mood scales after Sessions 1 and 3. On completion of the experiment, participants were led back downstairs for a short debrief.

### Data analysis

Questionnaire data were analysed using mixed-design repeated measures GLM (SPSS 23, IBM Corp. 2014) to establish the effects of room type (between-subjects factor: ‘Pleasant vs ‘Unpleasant’) and stage of experiment (within-subjects factor: baseline vs after Session 1 vs after Session 2) on the measures of participant mood. We predicted that mood would be negatively affected by the ‘Unpleasant’ room treatment. Data from Sessions 1 and 3 were analysed in two ways using Matlab. A model-agnostic analysis investigated the effects of room type on the probability of choosing SAFE or REW keys and on reaction time, according to the ambiguity of the presented stimulus (divided into bins of 0.2 ambiguity units in Session 3). A model-dependent analysis adopted the Bayesian decision model as described below.

#### Bayesian perceptual decision model

Whiteley and Sahani (2008) [24] described a Bayesian decision model for this problem. Consider a trial in which the true angle of the Gabor is *s* and the subject’s perceived value is *x*. In the model, it is assumed that *x* is drawn from a Gaussian distribution with mean *s* and standard deviation *σ*, $p\left(x|s\right)∼N\left(x;s,\sigma^{2}\right)$. This implies that the subject should calculate a posterior distribution over *s* given *x* according to Bayes rule

$$p\left(s|x\right)=\frac{p\left(x|s\right)p\left(s\right)}{p\left(x\right)}.$$ (1)

Here we assume that the prior distribution of *s*, *p*(*s*) is flat. Then $p(s|x)∼N\left(s;x,\sigma^{2}\right)$ is also a Gaussian. The ambiguity of the stimulus is thus determined by the signal to noise ratio *s*/*σ*. The model allows for the objective values for the win (*R*^+^) and loss (*R*^−^) to have subjective worth *c*^+^ *R*^+^ and *c*^−^ *R*^−^ respectively, where we introduced *c*^+^ and *c*^−^ as subjective sensitivity constants. The Bayesian optimal choice is then to choose the Risky key (rew) if the expected subjective value for doing so is greater than that for choosing the Safe key (safe; whose subjective value is just 0). Given *x*, the former is

$$E_{\text{REW}}\left(x\right)=c^{+}R^{+}P\left(s\geq 0|x\right)-c^{-}R^{-}P\left(s<0|x\right)$$ (2)

$$=c^{+}R^{+}\left(1-\Phi_{\sigma }\left(-x\right)\right)-c^{-}R^{-}\Phi_{\sigma }\left(-x\right)$$ (3)

where Φ_*σ*_(⋅) is the cumulative distribution function of a Gaussian with mean 0 and variance *σ*^2^. This is greater than 0 if

$$x>-\Phi_{\sigma }^{-1}\left(\alpha \right),\;\text{where}$$ (4)

$$\alpha =\frac{R^{+}}{R^{+}+C_{loss}R^{-}}$$ (5)

and $C_{\text{loss}}=\frac{c^{-}}{c^{+}}$ is the differential sensitivity of losses to wins. The participant knows *x*; however we do not. Thus we ask with what *probability* the participant will make the Risky choice given a true angle *s*. This is

$$P_{\text{REW}}=P\left(E_{\text{REW}}\left(x\right)>0|s\right)$$ (6)

$$=P\left(x>-\Phi_{\sigma }^{-1}\left(\alpha \right)|s\right)$$ (7)

$$=1-\Phi_{\sigma }\left(-(s+\Phi_{\sigma }^{-1}\left(\alpha \right))\right)$$ (8)

$$=\Phi_{\sigma }\left(s+\Phi_{\sigma }^{-1}\left(\alpha \right)\right)$$ (9)

To accommodate suboptimalities in subjects’ decisions, we followed [24] in introducing a systematic bias term *δ*, and a lapse rate *P*_Lapse_. The latter represents the probability of a perfectly uninformed decision *P*_REW_ = 0.5, thus encompassing all the fitting error of the model. In total, this implies

$$P_{\text{REW}}=\left(1-P_{\text{Lapse}}\right)\Phi_{\sigma }\left(s+\Phi_{\sigma }^{-1}\left(\alpha \right)+\delta \right)+\frac{P_{\text{Lapse}}}{2},$$ (10)

which contains four parameters: *σ*, *C*_loss_, *δ*, and *P*_Lapse_.

In Session 1, this model can be reduced to a two parameter model:

$$P_{\text{REW}}=\Phi_{\sigma }\left(s+\delta \right),$$ (11)

where we set *α* = 0.5 and *P*_Lapse_ = 0. Model comparison suggested that the lapse rate was not necessary for session 1 (see Table 1).

**Table 1:** iBIC scores for different models.

Session	Whiteley-Sahani Model	M	iBIC (Pleasant)	iBIC (Unpleasant)
1	Learn (*σ*, *δ*). Set *C*_loss_ = 1, *P*_Lapse_ = 0.	2	1117	954
1	Learn (*σ*, *δ*, *P*_Lapse_). Set *C*_loss_ = 1.	3	1132	967
3	Learn (*σ*, *δ*, *C*_loss_). Set *P*_Lapse_ = 0.	3	6125	6363
3	Learn (*σ*, *δ*, *C*_loss_, *P*_Lapse_).	4	6113	6352

#### Model fitting

Our primary goal was to determine the distribution of model parameters **h** for each group of participants. We therefore conducted a so-called Type II maximum likelihood or hierarchical Bayesian random effects analysis [25, 26]. In this, the (suitably transformed) parameters **h**_*i*_ of individual *i* are treated as a random sample from a population distribution, which we assume to be Gaussian with means and (diagonal) covariance ***θ*** = {***μ***_*θ*_, **Σ**_*θ*_}.

We determined the prior distribution ***θ*** using maximum likelihood:

$$\begin{array}{cc} \theta^{ML} & \approx {\text{argmax}}_{\theta }\left\{p\left(D|\theta \right)\right\}\\ & ={\text{argmax}}_{\theta }\left\{\prod_{i=1}^{N}\int dh_{i}\;p\left(D_{i}|h_{i}\right)p\left(h_{i}|\theta \right)\right\}, \end{array}$$ (12)

where **D** = {*D*_*i*_} comprises the data for all subjects, and *D*_*i*_ ∈ {SAFE, REW}. We found the maximum likelihood values for ***θ*** using an approximate Expectation-Maximization procedure. For the E-step of the k-th iteration, we assumed a Laplace approximation. As a result, we obtain,

$$m_{i}^{k}={\text{argmax}}_{h}\left\{p\left(D_{i}|h\right)p\left(h|\theta^{k-1}\right)\right\}\;\text{and}\;\text{thus}$$ (13)

$$p\left(h_{i}^{k}|D_{i}\right)\approx N\left(m_{i}^{k},\Phi_{i}^{k}\right),$$ (14)

where, according to a Laplace approximation, $N(m_{i}^{k},\Phi_{i}^{k})$ is a Normal distribution with mean $m_{i}^{k}$ and covariance $\Phi_{i}^{k}$ that is obtained from the inverse Hessian around $m_{i}^{k}$. For the M step:

$$\mu_{\theta }^{k+1}=\frac{1}{N}\sum_{i=1}^{N}m_{i}^{k},$$ (15)

$$\Sigma_{\theta }^{k+1}=\text{diag}\left\{\frac{1}{N}\sum_{i=1}^{N}\left(m_{i}^{k}{\left[m_{i}^{k}\right]}^{T}+\Phi_{i}^{k}\right)-\mu_{\theta }^{k+1}{\left[\mu_{\theta }^{k+1}\right]}^{T}\right\}.$$ (16)

The maximum a posteriori probability (MAP) estimate for each subject *i* is

$$m_{i}={\text{argmax}}_{h}\left\{p\left(D_{i}|h\right)p\left(h|\theta^{ML}\right)\right\}.$$ (17)

#### Model comparison

We compared models according to their integrated Bayes Information Criterion (iBIC) scores:

$$\text{iBIC}=-2\log p\left(D|\theta^{ML}\right)+M\log\left|D\right|,$$ (18)

where *M* is the number of fitted prior parameters (i.e., the length of **h**), and |**D**| is the number of data points (i.e., the total number of choices made by all participants), and

$$\log p\left(D|\theta^{ML}\right)=\sum_{i=1}^{N}\log\int dh\;p\left(D_{i}|h\right)p\left(h|\theta^{ML}\right)$$ (19)

$$\approx \sum_{i=1}^{N}\log\frac{1}{K}\sum_{j=1}^{K}p\left(D_{i}|h^{j}\right),$$ (20)

approximating the integral as an average over *K* samples **h**^*j*^’s generated from the prior *p*(**h**|***θ***^*ML*^).

There are various ways to test the hypothesis that the two groups of subjects are associated with different parameters. We compared a model employing just a single set of parameters to one with two sets, one each for Pleasant and Unpleasant Room subjects. The latter was preferred according to the iBIC scores for session 3 (Table 2).

**Table 2:** iBIC scores for separated groups and one group.

Session	Whiteley-Sahani Model	M	iBIC (separate)	iBIC (one group)
1	Learn (*σ*, *δ*). Set *C*_loss_ = 1, *P*_Lapse_ = 0.	2	2269	2052
3	Learn (*σ*, *δ*, *C*_loss_, *P*_Lapse_).	4	12265	12561

#### Model simulation

In order to see which aspects of data were captured by the model, we simulated the model with MAP estimate parameters for Session 1. The simulation was performed with the same angle conditions as in Session 1, and repeated 500 times for each participant. We also plotted the model’s outputs assuming the means of group parameters $\mu_{\theta }^{ML}$.

#### Permutation test

In permutation tests, data were randomly shuffled among subjects to create a new sample distribution. We then computed the value of current interest (e.g. the difference between the means of the Pleasant and the Unpleasant participants). This procedure was repeated many times (e.g. 10,000) to examine our null and alternative hypothesis (e.g. whether the difference is zero) by establishing where our observed difference lay on the re-sampled permutation distribution.

## Results

**Effects of room type on questionnaire data** The effects of room type on cross-time changes in measures of mood—Affect Grid Valence and Activation, and PA and NA—were analysed using mixed-design repeated-measures GLMs. Valence was not affected by room type (F(1, 46)<1) or time (F(2, 92) = 1.25, p = 0.293), and there was no interaction effect (F(2, 92)<1). There was a significant effect of time on Activation (F(1.7, 79.9) = 4.154, p = 0.024) and a time*room interaction (F(1.7, 79.9) = 4.12, p = 0.025), but no main effect of room (F(1, 46)<1) (S1a Fig). Activation increased as the experiment progressed in subjects in the Pleasant Room, but remained stable in subjects in the Unpleasant Room. PA was not affected by room (F(1, 46)<1) or time (F(1.5, 70.3) = 2.37, p = 0.114), and there was no interaction effect (F(1.5, 70.3) = 1.35, p = 0.262). There was a significant decrease in reported NA from the start to the end of the experiment (F(2, 92) = 13.34, p<0.001), and a non-significant trend for NA scores to fall more sharply in subjects in the Pleasant Room (time*room interaction effect (F(2, 92) = 2.59, p = 0.081)). There was no main effect of room (F(1, 46)<1) on NA scores (S1b Fig).

### Model-agnostic results

#### Session 1

**Fig 3:**
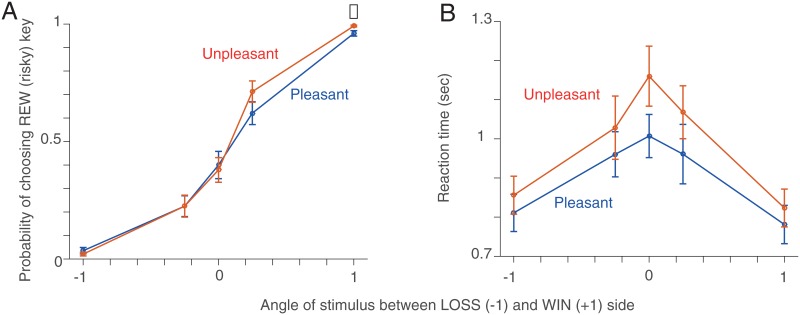
Model-agnostic results (Session 1). **A**. Probability of choosing the REW (risky) key in Session 1. The red (blue) line indicates the mean and the SEM of the probability of choosing the REW key in the Unpleasant (Pleasant) condition. When the stimulus strongly leaned toward the WIN side (*s* = 1), the difference between Pleasant and Unpleasant Room participants was mildly significant. **B**. Reaction time (RT). Both groups showed significant dependence of RT on stimulus ambiguity.

Most participants were risk-averse [t-test t(47) = 2.85, p = 0.006; sign test, p = 0.007], choosing the SAFE key significantly more often than the REW (risky) key when the stimulus was perfectly ambiguous *s* = 0. Both Pleasant and Unpleasant Room participant groups showed a similar choice preference. However, Pleasant Room participants were mildly more risk-averse (less risk-taking) than Unpleasant room participants [permutation test, p = 0.014] when the stimulus most strongly leaned toward the WIN side (*s* = 1; Fig 3A). However, when the stimulus leaned toward the LOSS side, we found no difference between two groups of participants.

As expected in perceptual decision-making tasks, participants’ reaction times (RTs) were significantly increased as stimulus ambiguity increased [Friedman’s test: Pleasant only *χ*^2^ = 43.07, *p* < 10^−7^, Unpleasant only *χ*^2^ = 45.43, *p* < 10^−8^], but there was no significant difference between Unpleasant and Pleasant Room participants (Fig 3B).

#### Session 3

**Fig 4:**
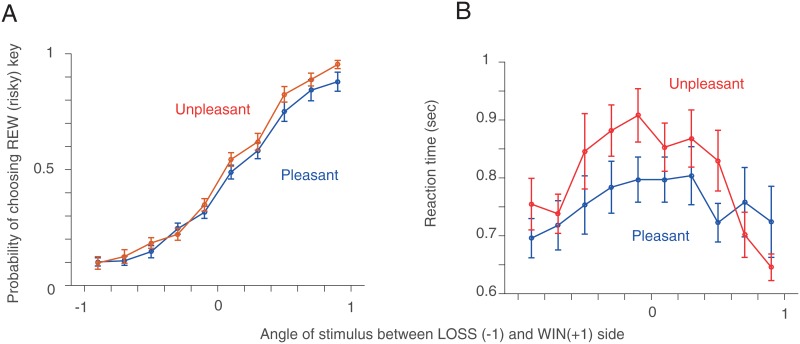
Model-agnostic results (Session 3). **A**. The probability of choosing the REW (risky) key in Session 3. The red (blue) line indicates the mean and the SEM of participants in the Unpleasant (Pleasant) condition. **B**. Reaction time (RT). Note that this session has 250 different stimulus ambiguities for each participants. We binned conditions with the width of *s* = 0.2.

Fig 4A and 4B show the probability of choosing the REW (risky) key and the reaction time in Session 3 in bins of stimulus ambiguity (*s* = 0.2), marginalizing over the different values of *R*^+^ and *R*^−^. We found that the difference in the probability of choosing the REW key between Unpleasant and Pleasant Room participants was not significant (Fig 4A). Both Pleasant and Unpleasant Room participants’ reaction time were significantly modulated by stimulus ambiguity [Friedman’s test: Pleasant only *χ*^2^ = 38.61, *p* < 10^−4^ Unpleasant only *χ*^2^ = 80.04, *p* < 10^−12^] (Fig 4B).

### Model-dependent results

There are various potential causes of the apparent choice bias seen in Fig 3. We therefore sought to use a formal characterization of decision-making to dissect potential cause, reveal what was influenced by Pleasant and Unpleasant Room conditions, and quantify the impact of the variation in *R*^+^ and *R*^−^ in Session 3. To do these, we fit a Bayesian perceptual decision making model to our data (see Methods) [24].

#### Session 1

**Fig 5:**
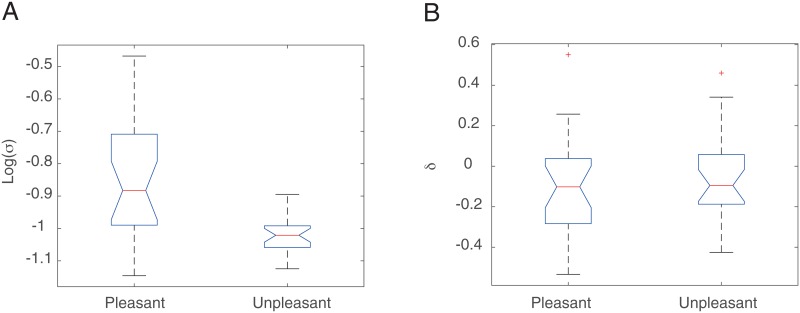
Estimated parameters of the two parameter model in Session 1. **A**. The noise parameter *σ*. The effect of Room manipulation (the difference between Pleasant and Unpleasant population) was significant. **B**. The bias parameter *δ*. The effect of Room manipulation (the difference between Pleasant and Unpleasant population) was not significant.

Planned model comparisons (see iBIC scores in Table 1) suggested that the lapse rate parameter *P*_Lapse_ was not necessary. The estimated parameters of the model without a lapse rate are shown in Fig 5. We found that subjects in the Pleasant Room had a significantly larger noise parameter *σ* than those in the Unpleasant Room [permutation test: mean *p* < 10^−4^, variance *p* < 10^−4^], but there was no difference between the rooms in the bias term (Fig 5A and 5B).

Although the iBIC score obtained by fitting data separately for Pleasant and Unpleasant Room groups did not show an improvement over the one obtained by fitting data as one group (Table 2), we found that the difference between the mean of the estimated parameter *σ* for Pleasant and Unpleasant groups was nearly significant even when fitting data as one group [permutation test, p = 0.052].

**Fig 6:**
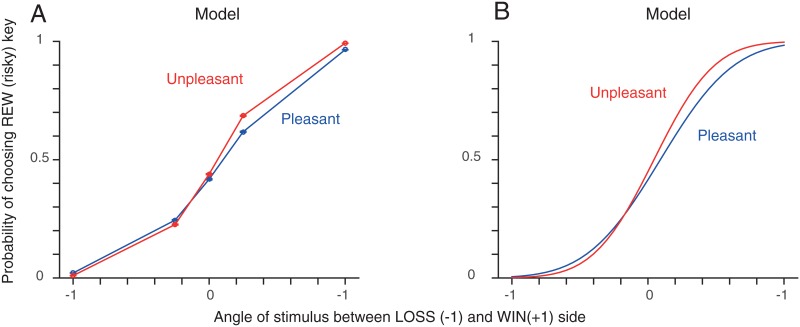
Model results for Session 1. **A**. Model simulation results with the estimated parameters ***m***_*i*_’s (Session 1). The model captures the asymmetry that is seen in the data (see Fig 3). The model was simulated in the same conditions (Session 1) as the actual experiment for each participant for 500 repetitions. The mean and the standard error of the mean of each participant group is shown. **B**. Model estimate with the estimated group means $\mu_{\theta }^{ML}$.

We also found that our model captured the asymmetric difference in choice between Pleasant and Unpleasant Room groups that we saw in the data. As seen in Fig 6, using the parameters determined from our model-fitting, simulations correctly reproduced the difference in choice between the two participant groups more strikingly on the WIN side than on the LOSS side (Fig 3A). This was because the cumulative Gaussian function in our model tends to express the difference more strongly at the opposite side to the overall bias. Since both groups had a bias toward the LOSS side, our model expressed the difference between groups more strongly on the WIN side, which is consistent with what we found in the data (Fig 3A)).

#### Session 3

**Fig 7:**
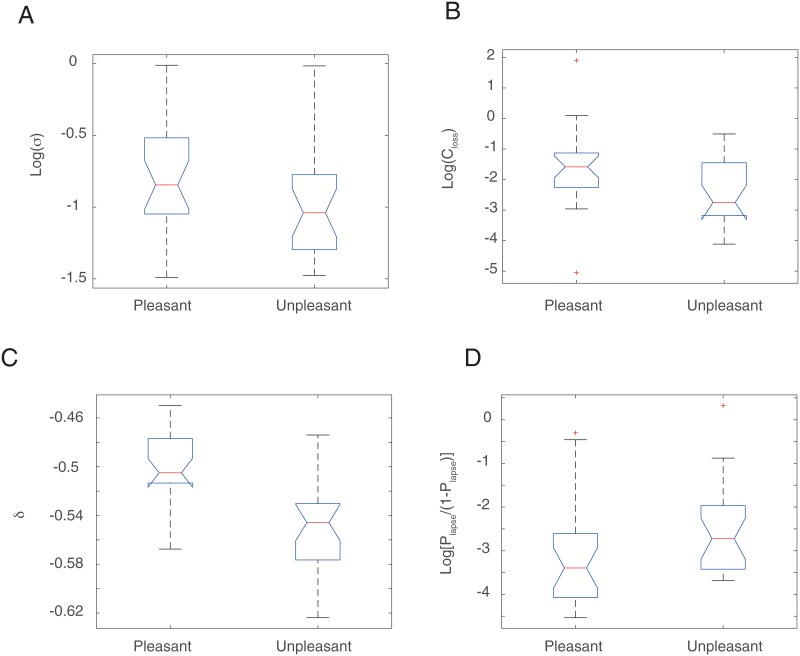
Estimated parameters of the four parameter model in Session 3. **A**. The noise parameter *σ*. The means of Pleasant and Unpleasant room participants were not significantly different. **B**. The relative weight of losses to wins. The means of Pleasant and Unpleasant room participants were significantly different. **C**. The bias parameter *δ*. The means of Pleasant and Unpleasant room participants were significantly different. **D**. The lapse rate. The means of Pleasant and Unpleasant room participants were mildly significantly different. The parameters are shown in the inference space.

Planned iBIC model comparisons suggested that all parameters of the Whiteley-Sahani model were important to fit data from Session 3 (Table 1); and furthermore that it was appropriate to fit Pleasant and Unpleasant Room groups separately (see Table 2). The estimated parameters are illustrated in Fig 7. The noise parameter did not differ significantly between the groups in this session (Fig 7A). However, losses were significantly more heavily weighted than wins (*C*_Loss_ < 1; Fig 7B), to a greater degree for Pleasant Room participants [permutation test, p = 0.018]. Further all participants were biased toward the SAFE key (*δ* < 0; Fig 7C) to a degree that was significantly larger for Unpleasant Room participants [permutation test, p = 0.0079]. This means that Unpleasant Room participants were more strongly driven by wins relative to losses compared to Pleasant Room participants, but they also showed a stronger bias towards choosing the SAFE key. The lapse rate for Pleasant room participants was mildly smaller than Unpleasant room participants [permutation test, p = 0.046; Fig 7D].

**Fig 8:**
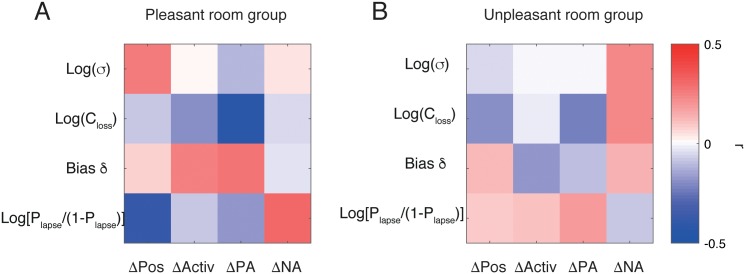
Correlations between estimated parameters and the changes in questionnaire scores (affective measures). **A** The Pleasant room participants. **B**. The Unpleasant room participants. The colour shows the correlation coefficient *r*, where blue means negative (*r* < 0) and red means positive (*r* > 0). The changes in score were computed as the score after Session 3 minus the score before Session 1. None of the correlations was significant after the multiple comparison correction.

Fig 8 shows how our model parameters were correlated with scores on the mood questionnaires. We defined changes in questionnaire scores as the questionnaire score after the task minus the questionnaire score before the task.

## Discussion

The goal of this study was to dissect how affect manipulations influence computations that underlie decision-making under ambiguity. To achieve this we employed a judgement bias task, developed to investigate the influence of affect on decision-making in non-human animals and back-translated for use with humans. We added trial-by-trial variations in the offered rewards and threatened punishments to the conventional variations in perceptual ambiguity, allowing us to probe how decisions were affected by these different factors and their interactions, and how this was influenced by manipulations of affective state. We used both model-agnostic analyses and an approximate Bayesian decision-theoretic model [24] to test which aspects of computation during decision-making (e.g. perception, valuation, bias) were influenced by our affect manipulation.

Participants were assigned to either a Pleasant or Unpleasant room. This led to some differences in questionnaire measures of affective state across the study. Pleasant Room subjects showed an increase in the Affect Grid measure of Activation across time which was not observed in subjects in the Unpleasant Room. This was more evident during Session 1. There was also a non-significant trend (p<0.1) for Pleasant but not Unpleasant room subjects to show a decrease in the PANAS scale measure of negative affect (NA) across test sessions. Overall, however, effects were very small even though similar affect manipulations have been used successfully in the past (sweet gift: [23, 27]); future studies might employ alternative manipulations such as use of affective images and music, and recall of affectively salient events [28]. In order to minimise the chances of subjects conforming to their expectations of how an emotion manipulation should affect their behaviour, we deliberately attempted to impose an implicit manipulation by not informing them about the presence of the two different rooms. It would be interesting to compare the effectiveness of implicit and explicit manipulations in future studies. Nevertheless, despite the small changes in questionnaire responses, clear changes in decision-making were detected raising the possibility that alterations in choice behaviour may sometimes be more sensitive to affective state than questionnaire measures of self-reported affect, and that this may be especially likely when affect is implictly manipulated (cf. [29]).

It was in Session 3 that the various conditions were altered on a trial-by-trial basis in accordance with the main aims of the study. A model-agnostic analysis investigated the effect of stimulus ambiguity on the probability of choosing the REW key, and reaction times. No significant effect of treatment on REW key choice were observed, but reaction time increased with increasing stimulus ambiguity, as is standard for simple perceptual decisions [30]; there was no effect on this of the room. A model-based analysis incorporated the influence of trial-by-trial variation in prospective WINS or LOSSES. The participants were biased towards choosing the SAFE key, reflecting risk aversion under ambiguity. This is consistent with loss aversion (for example, when people are faced with a gamble in which there is a 50:50 chance of winning or losing the same amount of money (e.g. [31, 32]). However, subjects in the Unpleasant Room were more biased in this direction than those in the Pleasant Room. At the same time, they weighed wins more heavily than losses. There was no strong correlation between estimated model parameters for each individual and psychometric measures of their mood.

The stronger pessimistic (risk-averse) bias observed in Unpleasant Room subjects during Session 3 is consistent with our prediction that the Unpleasant Room manipulation should lead to more pessimistic decision-making under ambiguity, even though we found only weak evidence, using the PANAS scale, of more reported negative affect in the Unpleasant Room. The finding is also in line with other studies of affect-induced judgement biases in both humans [15–17] and animals [7, 33–37].

Alongside their more pessimistic bias, Unpleasant Room subjects weighed wins more heavily than losses relative to Pleasant Room subjects. These findings appear to contradict each other; it might be expected that risk aversion, as observed in Unpleasant Room subjects, would be associated with a greater valuation of loss relative to win. However, it is conceivable that the findings are caused by two different underlying processes associated with a more negative affective state. First, negative mood states may reflect previous experience of an elevated rate of negative events, and act as a Bayesian prior that the probability of future negative events is high [9, 38, 39]. This results in an adaptive enhanced expectation of negative outcomes under ambiguity leading to risk-averse, or pessimistic, decisions. At the same time, however, there are data suggesting that mild negative mood states may be associated with an increase in reward valuation [40–44], perhaps because acquiring available rewards can help to enhance mood state; a form of mood repair [43, 45]. Longer-term and deeper negative moods may, on the other hand, be associated with devaluation of reward as evidenced by anhedonic responses [46], perhaps functioning to minimise activity and associated risks and energy expenditure [47]. Thus a mild negative mood might be characterised by an increased perceived probability of negative things happening, but also an increased valuation of available reward, as appears to be suggested by our findings. This intriguing possibility requires more investigation using techniques such as those developed here, coupled with behavioural tasks and/or psychometric instruments that can separately measure expected probabilities and reward valuation.

The version of the task in Session 1 is more directly comparable to that used in animal studies in which outcomes of decisions are fixed throughout; offered WINS and threatened LOSSES were +50p and -50p in all trials. In our model-agnostic analysis, we found that subjects from both groups were risk averse under ambiguity, preferring to choose the SAFE key when presented with a completely ambiguous stimulus, as in Session 3. When the stimulus leant towards the WIN side, but not when it leant towards the LOSS side, Unpleasant Room subjects chose the REW key more often than Pleasant Room subjects. Our Bayesian model captured this asymmetry effect, without appealing to the possibility that subjective values are non-linear functions of WIN and LOSS [31]. This was because the Bayesian decision-maker created a non-trivial dependence of choice on the bias, such that any difference in choice was more strongly expressed at the opposite side to the overall bias which, in our study, was towards the LOSS side. This implies that other known asymmetric effects could also be accounted for by such a Bayesian computation, rather than by appealing to heuristics. The model-dependent analysis also revealed that Pleasant Room subjects had a larger and more variable noise parameter than Unpleasant Room subjects, indicating that they were less accurate (sensitive) in their classification of stimuli as leaning to the WIN or LOSS side. No treatment effect on reaction times was observed, but, as in Session 3, these increased as stimulus ambiguity increased.

The greater propensity for Unpleasant Room subjects to choose the REW key when the stimulus leant towards the WIN side (an optimistic response) detected in our model-agnostic analysis contrasts with our predictions and the findings from Session 3, and with several other studies using the judgment bias paradigm in humans [15–17]. However, it is consistent with the the finding that Pleasant Room subjects were more noisy in their choices, indicating greater inaccuracy when responding to less ambiguous cues, and thus a relatively lower likelihood of pressing the REW key when the stimulus was leaning towards the WIN side. However, we would also have expected to see a lower likelihood of pressing the SAFE key when the stimulus leant towards the LOSS side, and this was not observed The reasons for the treatment effect on the noise parameter is unclear. One speculative possibility is that the apparent rise in the Affect Grid measure of Activation in Pleasant relative to Unpleasant Room subjects during Session 1 was associated with decreased attention to the task, but independent evidence for such an effect is lacking as is any reason for why it should be expressed in response to stimuli leaning towards the WIN side only.

In previous animal studies, asymmetrical results of this sort have also been observed. For example, rats in unpredictable (stressful) housing conditions were less likely and slower than those in predictable housing to make a response indicating anticipation of reward when exposed to auditory tones close to a trained tone that predicted reward, but not when exposed to tones close to those that predicted punishment [7]. In contrast, Burman et al. [48] found the opposite asymmetry, observing differences in decision-making between rats housed in enriched and unenriched conditions when exposed to cues close to a location that predicted no reward but not when exposed to those close to a location that predicted reward. However, it is difficult to compare these findings directly with our current results because, in animal studies, choice outcomes are often provided in different ‘currencies’ (e.g. food vs noise) in contrast to the single currency used here (win 50p vs lose 50p). The intrinsic rewarding or aversive values of these different currencies may be an important influence on which ‘ambiguous’ cues reveal biases [10], that is not present in human studies.

In summary, we have introduced a new variant of the judgement bias task in which trial-by-trial variation of variables that influence decision-making can, in combination with the use of Bayesian decision models, be used to tease apart processes underlying the impact of affective states on decision-making under ambiguity. In future, and given clever experimental designs that are feasible to train, it may be possible to implement such tasks in animals. However, at present, studies of humans may provide novel insights into decision-making processes that could explain variation in findings from animal research. For example, the possibility, raised in this study, that affective states may have different influences on perceived probability and valuation of rewards (and punishments) may be one explanation for such inconsistencies.

There are various appealing directions for future studies. One would be to attempt a counter-balanced go/no-go version of the task rather than the go/go version used here (i.e. requiring subjects to press or withhold pressing a single manipulandum rather than choosing which one of two levers to press). This would then implement a version of the orthogonalized go/nogo; reward/punishment task studied by [49, 50]. This revealed various interactions between instrumental and Pavlovian influences over action, and it would be possible to test the hypothesis that mood acts through the latter. A second direction would be to use aversive stimuli as the consequence of a failed risky choice and then to manipulate anxiety in order to see whether similar influences of affect on decision-making processes are observed in the context of punishment. A third would be to explore the influence of instruction in creating the balance between the implicit desire of subjects to get the perceptual answer correct, versus maximizing their gains or minimizing their losses. A fourth would be to compare the effects of implictly or explictly imposed affect manipulations, as discussed earlier.

## Supporting Information

S1 Fig Changes in mean (+/- SEM) (a) Affective Grid Activation score and (b) PANAS NA score across the study in Pleasant Room (solid line) and Unpleasant Room (dashed line) subjects. (EPS)
